# Fast Three-Dimensional Profilometry with Large Depth of Field

**DOI:** 10.3390/s24134037

**Published:** 2024-06-21

**Authors:** Wei Zhang, Jiongguang Zhu, Yu Han, Manru Zhang, Jiangbo Li

**Affiliations:** 1 Department of Computer Technology and Science, Anhui University of Finance and Economics, Bengbu 233030, Chinam17375254816@163.com (M.Z.); 2 College of Intelligent Manufacturing, Foshan Polytechnic, Foshan 528137, China; zhujguang95@163.com; 3 Intelligent Equipment Research Center, Beijing Academy of Agriculture and Forestry Sciences, Beijing 100097, China

**Keywords:** three-dimensional profilometry, large depth of field, time-domain Gaussian fitting, neural network

## Abstract

By applying a high projection rate, the binary defocusing technique can dramatically increase 3D imaging speed. However, existing methods are sensitive to the varied defocusing degree, and have limited depth of field (DoF). To this end, a time–domain Gaussian fitting method is proposed in this paper. The concept of a time–domain Gaussian curve is firstly put forward, and the procedure of determining projector coordinates with a time–domain Gaussian curve is illustrated in detail. The neural network technique is applied to rapidly compute peak positions of time-domain Gaussian curves. Relying on the computing power of the neural network, the proposed method can reduce the computing time greatly. The binary defocusing technique can be combined with the neural network, and fast 3D profilometry with a large depth of field is achieved. Moreover, because the time–domain Gaussian curve is extracted from individual image pixel, it will not deform according to a complex surface, so the proposed method is also suitable for measuring a complex surface. It is demonstrated by the experiment results that our proposed method can extends the system DoF by five times, and both the data acquisition time and computing time can be reduced to less than 35 ms.

## 1. Introduction

Quickly and accurately acquiring a 3D point cloud of an object’s surface is important in numerous fields, such as quality control, robotic assembly, medical treatment, virtual reality, and reverse engineering [1,2,3]. With the advantages of non-contact, high speed, and high accuracy, fringe projection profilometry (FPP) has become one of the most promising 3D imaging techniques. In conventional FPP system, a set of 8-bit sinusoidal patterns will be projected onto the object surface. As an 8-bit gray pattern has limited projection rate (within 120 Hz), the measurement speed of FPP system is thus restricted [4].

By applying 1-bit binary patterns which have much higher projection rates (up to 20 kHz), the binary defocusing technique can greatly improve 3D imaging speed [5,6,7]. The squared binary defocusing method (SBM) is the simplest binarization strategy, which utilizes the binary patterns with the shape of square wave to create sinusoidal fringes [8]. Conventional binary defocusing techniques require a proper defocusing degree to achieve ideal sinusoidal fringes, otherwise significant measurement errors may arise. It thus is sensitive to the defocusing degree and has a small DoF. Various advanced binarization strategies have been proposed to enhance DoF, such as the sinusoidal pulse width modulation (SPWM) [9], optimal pulse width modulation (OPWM) [10], and the dithering method [11]. As these methods still require a proper defocusing degree for generating sinusoidal fringes, their enhancements in DoF are rather limited.

Many methods were introduced to minimize the measurement errors which are caused by using improper defocusing degrees. After projecting the 8-bit gray patterns and binary patterns to a white board, respectively, Xu et al. obtained the phase error distribution in a large depth range and then built a mathematical model to eliminate the phase error at arbitrary depth ranges [12]. Hu et al. utilized the depth-discrete Fourier series fitting to reduce the complexity of the phase error model [13]. In Zhu’s model, more influence factors were taken into account (including defocusing level, intensity noise, and fringe frequency), and the optimal fringe frequency of the binary error-diffusion fringe pattern can be selected [5]. Yu et al. achieved an accurate 3D reconstruction in a large DoF by directly transforming the captured patterns into the desired phase with deep learning models [14]. Although these methods may work well in error compensation, however, it is a tedious process to collect accurate phase errors in a large DoF, and expensive equipment is also required.

In this paper, a time-domain Gaussian fitting method is proposed to suppress sensitivity of defocusing degree. Different from the phase-shifting algorithm, projector coordinates can be achieved by projecting Gaussian fringes and determining the peak positions of time-domain Gaussian curves. The neural network technique is applied to rapidly compute peak positions of time-domain Gaussian curves. Finally, by generating Gaussian fringes with defocused binary patterns, the time-domain Gaussian fitting method can be combined with the binary defocusing technique. The high projection rate can be then applied in FPP with much lower sensitivity of defocusing degree, which helps to achieve fast three-dimensional profilometry with a large DoF.

The centerline extraction technique is also adopt in line structured light [15] and multi-line shift profilometry [16], and lots of algorithms have been proposed, such as the Steger algorithm [17] and Skeleton extraction method [18]. However, these algorithms work with the spatial distribution of Gaussian fringes, which are modulated by the object’s surface and may deform accordingly. This will cause difficulty in obtaining accurate measurements of complex surfaces. Comparatively, as the time-domain Gaussian curve is extracted from an individual image pixel, its shape will not deform according to the complex surface. This is beneficial to acquire accurate measuring results of complex surfaces.

Compared with the traditional strategy, which imitates sinusoidal stripes with a proper defocusing degree, Gaussian stripes can be easily generated with a simple binary pattern. Different from extracting phase information with sinusoidal stripes, the peak positions of Gaussian stripes are the key information for 3D scanning. Although the varied defocusing degree may lead to a variation in the blur radius of Gaussian stripes, the peak positions of Gaussian fringes, however, will keep fixed. The varied defocusing degree thus may have much less of an impact on the proposed method. Although the neural network technique can be use to reduce the computing time, the calculation process of extracting peak positions of time-domain Gaussian curves is indeed more complex than that of the calculating phase with sinusoidal stripes.

The rest of this paper is organized as follows. The principle of time-domain Gaussian fitting method is explained in Section 2. The neural network-based rapid calculation approach is stated in detail in Section 3. Sensitivities to defocusing degree and complex surface are analyzed, respectively, in Section 4. The performance of the proposed method is verified in Section 5, and its characters are summarized in Section 6.

## 2. Principle

### 2.1. Determining the Projector Coordinate with a Time-Domain Gaussian Curve

**Figure 1:**
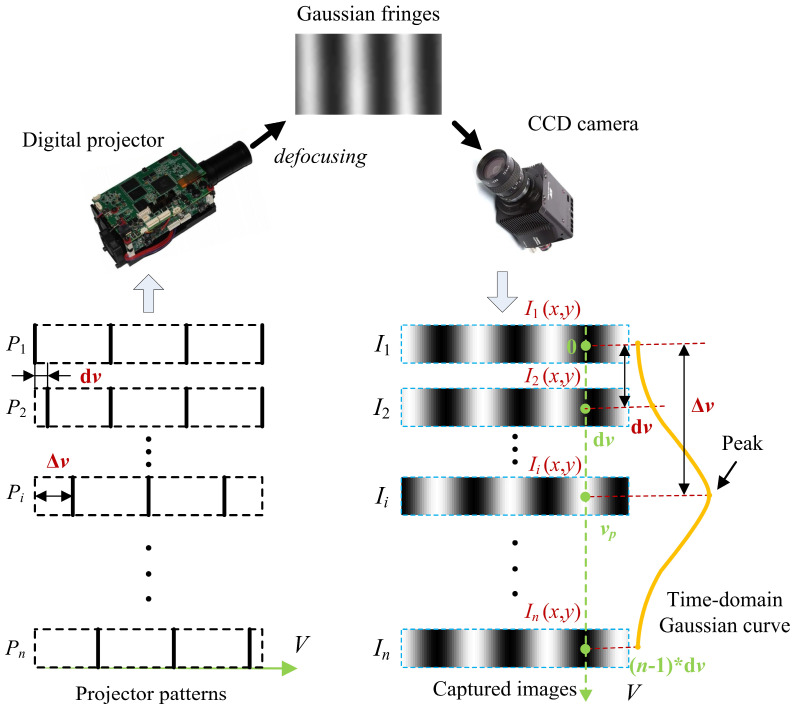
Schematic diagram of determining the projector coordinate.

The multi-line binary patterns (*P*_1_, *P*_2_, ···, *P*_n_) with uniform intervals are designed to generate the Gaussian fringes. In the multi-line binary patterns, the lines will gradually shift to a specific distance (d*v*) along the projector axis *V.* The interval between the two lines is equal to the product of the distance of the shifting step (d*v*) and the number of shifting steps (*n*). By defocusing the binary multi-line patterns, evenly spaced Gaussian fringes can be created to illuminate the objects. As shown in Figure 1, when the multi-line binary patterns are sequentially projected by the digital projector, the generated Gaussian fringes also shift with the constant speed, and the images of the Gaussian fringes (*I*_1_, *I*_2_, ···, *I_n_*) can be captured simultaneously. With respect to the image coordinate (*x*, *y*), intensity sequences *I*_i_ (*x*, *y*) are the uniform sampling of the Gaussian fringe, and can form a time–domain Gaussian curve. 

Because the distance of the shifting steps of multi-line patterns are identical and go along the projector axis *V*, the projector coordinate, *v*, can be set as the horizontal axis of the time–domain Gaussian curve (see Figure 1). Suppose that a line in the projector pattern has shifted the distance of Δ*v*, the Gaussian fringe will move simultaneously, and the intensity of the time–domain Gaussian curve just reaches the highest value (the peak of the time–domain Gaussian curve). By this time, the projector coordinate of the line is corresponds to the image coordinate (*x*, *y*). There, the shifting distance, Δ*v*, can be seen as the relative projector coordinate of the line. While the initial coordinate of the line is set to zero, its relative projector coordinate is equal to the peak position of the time–domain Gaussian curve *v_p_* (Δ*v = v_p_*). Therefore, the projector coordinate corresponding to image coordinate (*x*, *y*) can be determined by finding the peak position of the time–domain Gaussian curve.

In this paper, the time–domain Gaussian curve is modeled as a one-dimensional Gaussian function:

(1) $$G\left(\lambda ,\eta ,\sigma ,v_{p};v\right)=\lambda +\eta *\exp\left[-\frac{{\left(v-v_{p}\right)}^{2}}{2\sigma^{2}}\right]$$

where *λ* represents the bias, *η* denotes the scale factor, *σ* denotes the variance, and *v_p_* is the peak position of the time–domain Gaussian curve.

Peak position, *v_p_*, can be determined by finding the optimal value of following objective function with the Levenberg–Marquardt algorithm [19]:

(2) $$\min{\sum_{i=1}^{n}\left[G\left[\lambda ,\eta ,\sigma ,v_{p};v_{i}(x,y)\right]-I_{i}\left(x,y\right)\right]}^{2}$$

where *n* represents the number of captured images, *v_i_* denotes the abscissa values of the time–domain Gaussian curve, and *x* and *y* indicate the coordinates in both directions on the image plane. Because Equation (1) contains four undetermined parameters (*λ*, *η*, *σ*, and *v_p_*), at least four elements should be included in the time–domain Gaussian curve to yield a reliable result. This means that the number of captured images should be no less than 4 (n≥4). 

In the proposed method, as the binary lines are evenly spaced in the projector pattern, the maximum value of the relative projector coordinate, Δ*v*, will be restricted by the distance between two adjacent lines. Just like the wrapped phase map in the phase-shifting method [4], the relative projector coordinates Δ*v* (*x* and *y*) also can be converted into the absolute projector coordinate *v* (*x* and *y*) using the phase-unwrapping method [20]:

(3) v(x,y)=D∗C(x,y)+Δv(x,y)

where *D* is the distance between two adjacent lines in projector pattern, and *C* (*x* and *y*) represents the coded values for phase unwrapping.

### 2.2. Polynomial 3D Reconstruction Model

In an FPP system, a 3D reconstruction model is required to convert the distribution of projector coordinates into 3D coordinates. Among the existing 3D reconstruction models, the polynomial reconstruction model is more flexible to take nonlinear factors (such as lens distortion in the camera and projector) into account [21]. Although the polynomial reconstruction model with higher order is more accurate, it is prone to be ill conditioned if the order is higher than three [21]. Therefore, a third-order polynomial model is employed in this work, which can be formulated as the following:

(4) $$\{\begin{array}{c} \begin{array}{l} X=a_{1}+a_{2}x+a_{3}y+a_{4}v+a_{5}\mathrm{xy}+a_{6}\mathrm{xv}+a_{7}\mathrm{yv}+a_{8}x^{2}+a_{9}y^{2}+a_{10}v^{2}+a_{11}x^{2}y\\ +a_{12}x^{2}v+a_{13}{\mathrm{xy}}^{2}+a_{14}y^{2}v+a_{15}{\mathrm{xv}}^{2}+a_{16}{\mathrm{yv}}^{2}+a_{17}\mathrm{xyv}+a_{18}x^{3}+a_{19}y^{3}+a_{20}v^{3} \end{array}\\ \begin{array}{l} Y=b_{1}+b_{2}x+b_{3}y+b_{4}v+b_{5}\mathrm{xy}+b_{6}\mathrm{xv}+b_{7}\mathrm{yv}+b_{8}x^{2}+b_{9}y^{2}+b_{10}v^{2}+b_{11}x^{2}y\\ +b_{12}x^{2}v+b_{13}{\mathrm{xy}}^{2}+b_{14}y^{2}v+b_{15}{\mathrm{xv}}^{2}+b_{16}{\mathrm{yv}}^{2}+b_{17}\mathrm{xyv}+b_{18}x^{3}+b_{19}y^{3}+b_{20}v^{3} \end{array}\\ \begin{array}{l} Z=c_{1}+c_{2}x+c_{3}y+c_{4}v+c_{5}\mathrm{xy}+c_{6}\mathrm{xv}+c_{7}\mathrm{yv}+c_{8}x^{2}+c_{9}y^{2}+c_{10}v^{2}+c_{11}x^{2}y\\ +c_{12}x^{2}v+c_{13}{\mathrm{xy}}^{2}+c_{14}y^{2}v+c_{15}{\mathrm{xv}}^{2}+c_{16}{\mathrm{yv}}^{2}+c_{17}\mathrm{xyv}+c_{18}x^{3}+c_{19}y^{3}+c_{20}v^{3} \end{array} \end{array}$$

where, ***X***, ***Y*,** and ***Z*** denote the 3D coordinate vectors, and (*a*_1_, *a*_2_, ···, *a*_20_), (*b*_1_, *b*_2_, ···, *b*_20_), and (*c*_1_, *c*_2_, ···, *c*_20_) represent the coefficients of the polynomial model.

In general, the coefficients of the polynomial 3D reconstruction model can be calibrated with the least-squares algorithm [22]. The calibration data can be obtained by using the planar target and Zhang’s method [23].

## 3. Rapid Calculation Method

**Figure 2:**
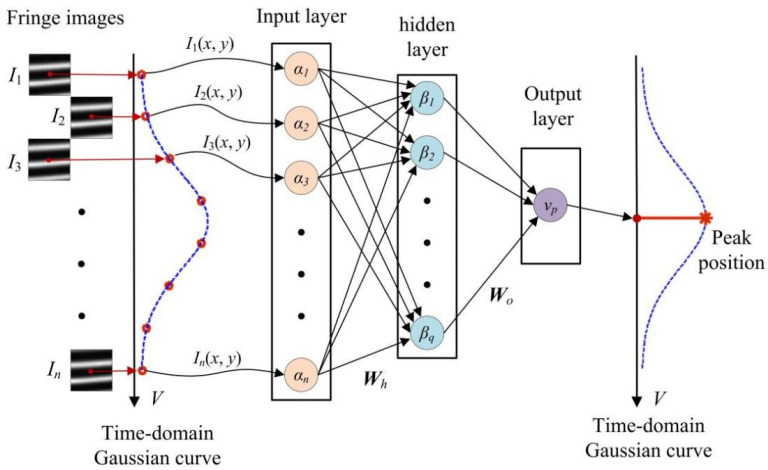
Principle of computing peak positions of time-domain Gaussian curves with the neural network.

Since the calculation process of the Levenberg–Marquardt algorithm involves iterative optimization, it may yield accurate peak positions, as well as causes low computational efficiency. To address this issue, a neural network-based approach is proposed to rapidly extract peak positions of time–domain Gaussian curves. The basic principle of this neural network-based approach is shown in Figure 2.

The proposed neural network consists of an input layer, an output layer, and a hidden layer. The intensity sequence, *I*_i_ (*x*, *y*) (i=1,2,⋯,n), is taken as the input of the neural network. The number of neurons in input layer is *n*, and the output of this layer is (*α*_1_, *α*_2_, ···, *α_n_*). The hidden layer contains *q* neurons, and yields the result (*β*_1_, *β*_2_, ···, *β_q_*). The output layer finally exports the peak position *v_p_* of the time–domain Gaussian curve. The weight matrix from the input layer to the hidden layer is ***W****_h_*, and ***W****_o_* represents the weight matrix from the hidden layer to the output layer. 

**Figure 3:**
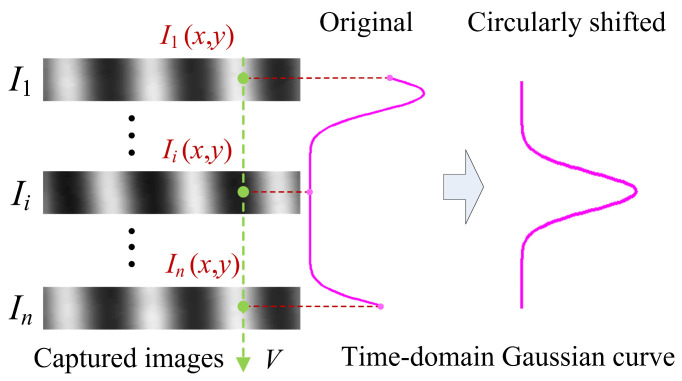
Schematic diagram showing the circularly shifting of the time-domain Gaussian curves.

Actually, most time–domain Gaussian curves are the sampling results of two adjacent Gaussian fringes. They cause the cyclic shift in the time–domain Gaussian curves, as shown in Figure 3. For this reason, while the time–domain Gaussian curve shifts continuously, the values of peak positions, however, have mutations in the edge region. This discontinuous correspondence would lead to a difficultly in computing accurate peak positions with the neural network.

Therefore, before taking it to be the input data of the neural network, the time–domain Gaussian curve should be preprocessed with additional circular shifting (see Figure 3). The shifting distance *d*_s_ (*x*, *y*) can be approximately estimated by subtracting the position of the maximum value of the time–domain Gaussian curve *v_max_* (*x*, *y*) from the middle position, $v_{mid}$.

(5) $$d_{s}\left(x,y\right)=v_{mid}-v_{max}\left(x,y\right)$$

**Figure 4:**
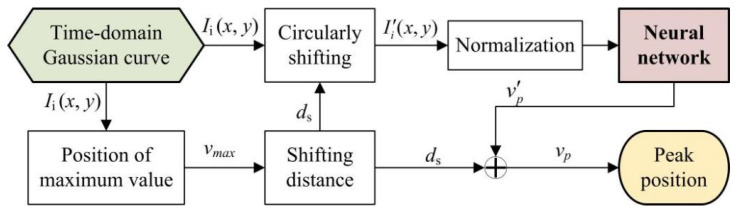
Flow chart of computing peak positions with preprocessing procedures.

With additional circular shifting, the peak position of the time–domain Gaussian curve will be changed to the middle area (see Figure 3). The discontinuous correspondence in the edge region can be avoided. The practical process of computing peak positions with the neural network is shown in Figure 4. Since the neural network merely yields the peak positions of circularly shifted Gaussian curves ${v^{\prime }}_{p}$, the actual peak positions, *v_p_*, can be achieved by adding the shifting distance, *d*_s_ ($v_{p}={v^{\prime }}_{p}+d_{s}$). 

In order to determine the parameters of the neural network, the training data can be obtained using the Levenberg–Marquardt algorithm. While applying this algorithm, initial values may significantly influence computing efficiency. It is recommended that the minimum value *v_min_*, the maximum value *v_max_*, and the middle position *v_mid_* of the circularly shifted time–domain Gaussian curve can be applied as the initial values of *λ*, *η*, and *v_p_* in Equation (1). 

## 4. Characteristics Analysis

### 4.1. Formatting of Mathematical Components

The Gaussian fringes are generated by defocusing the binary multi-line patterns, and the process of optical defocusing blur can be described as the following:

(6) $$F\left(u,v\right)=M\left(u,v\right)⊗h\left(u,v\right)=\int_{-\infty }^{+\infty }\int_{-\infty }^{+\infty }M\left(s,t\right)*h\left(u-s,v-t\right)dsdt$$

where *M* (*u*, *v*) and *F* (*u*, *v*) represent the multi-line pattern and defocused patterns, respectively, *h* is the defocusing PSF, ⨂ denotes the convolution operator, *u* and *v* indicate the coordinates in both directions on the defocused pattern (also the projector plane), and *s* and *t* present the coordinates in both directions on the multi-line pattern.

For convenience, the analysis process is carried out in one-dimensional space (projector axis *V*). In the first process, the defocusing of PSF *h* can be modeled as a one-dimensional Gaussian function:

(7) $$h\left(v\right)=\frac{1}{\sqrt{2\pi }\sigma_{h}}*\exp\left[-\frac{v^{2}}{2{\sigma_{h}}^{2}}\right]$$

where *σ_h_* denotes the blur radius which is related to the defocusing degree.

**Figure 5:**
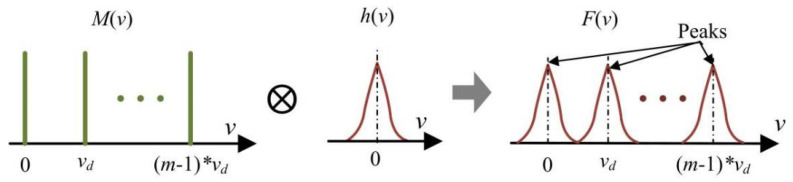
The Gaussian fringes are generated by blurring the multi-line pattern (in one-dimensional space).

In one-dimensional space (projector axis *V*), the multi-line pattern can be described as a set of Dirac delta functions (as shown in Figure 5):

(8) $$M\left(v\right)=\sum_{k=0}^{m-1}\delta \left(v-k*v_{d}\right)$$

where *m* is the number of lines in projector pattern, and *v_d_* represents the distance between two adjacent lines.

After the one-dimensional convolution operation, the defocused pattern in one-dimensional space *F* (*v*) can be described as the following:

(9) $$\begin{array}{ll} F\left(v\right)=M\left(v\right)⊗h\left(v\right) & =\sum_{k=0}^{m-1}\int_{-\infty }^{+\infty }\delta \left(v-k*v_{d}\right)*h\left(v-t\right)dt\\ & =\sum_{k=0}^{m-1}h\left(v-k*v_{d}\right)\\ & =\sum_{k=0}^{m-1}\frac{1}{\sqrt{2\pi }\sigma_{h}}*\exp\left[-\frac{{\left(v-k*v_{d}\right)}^{2}}{2{\sigma_{h}}^{2}}\right] \end{array}$$

As illustrated in Equation (9) and Figure 5, the variation in blur radius, *σ_h_* (corresponding to the varied defocusing degree), does not change the peak positions of the Gaussian fringes (in defocused pattern) as well as the time–domain Gaussian curves. It means that a varied defocusing degree theoretically has little impact on the proposed method, which achieves the projector coordinates by finding the peak position of the time–domain Gaussian curves. 

In spite of this, the calculation accuracy of peak positions may be influenced by the distance between two adjacent lines (*v_d_*). When the distance is too small, there exists an overlap between the adjacent fringes, which may lead to low contrast and high noise in the captured images. This will reduce the calculation accuracy of the proposed method. Moreover, it also makes it difficult to accurately unwrap the relative projector coordinates (peak positions).

Comparatively, the phase-shift algorithm [24] determines projector coordinates by calculating the phase of sinusoidal fringes. When they are generated by defocusing binary patterns, ideal sinusoidal fringes only can be achieved with a specific defocusing degree. As the defocusing degree varies in the whole DoF, ideal sinusoidal fringes thus exist in a small range in DoF. In another range in DoF, a nonsinusoidal fringe can be observed and taken to be a combination of an ideal sinusoidal fringe and high-order harmonics [25].

(10) $$I_{si}\left(x,y\right)=\omega_{0}+\sum_{\zeta =1}^{\infty }\omega_{\zeta }\left\{\zeta \left[\phi \left(x,y\right)+\delta_{i}\right]\right\}$$

where $I_{si}$(i=1,2,⋯,N) is the image of sinusoidal fringe, and *N* represents shifting number, ($\omega_{0},\omega_{1},\cdots ,\omega_{\zeta }$) are constants, ϕ(x,y) is the phase, and $\delta_{i}$ is the phase-shifting amount.

With the high-order harmonics, the computed phase deviates from the ideal phase value. The phase error can be expressed as the following:

(11) $$\Delta \phi \left(x,y\right)=\arctan\left[\frac{\sum_{\zeta =1}^{\infty }\left\{\left(\omega_{\zeta N+1}-\omega_{\zeta N-1}\right)\sin\left[\zeta N\phi \left(x,y\right)\right]\right\}}{\omega_{1}+\sum_{\zeta =1}^{\infty }\left\{\left(\omega_{\zeta N+1}+\omega_{\zeta N-1}\right)\cos\left[\zeta N\phi \left(x,y\right)\right]\right\}}\right]$$

As shown in Equation (11), a varied defocusing degree will lead to a periodic phase error in the results of the phase-shift algorithm, which finally can reduce the accuracy of the FPP system.

### 4.2. Sensitivity to Complex Surface

**Figure 6:**
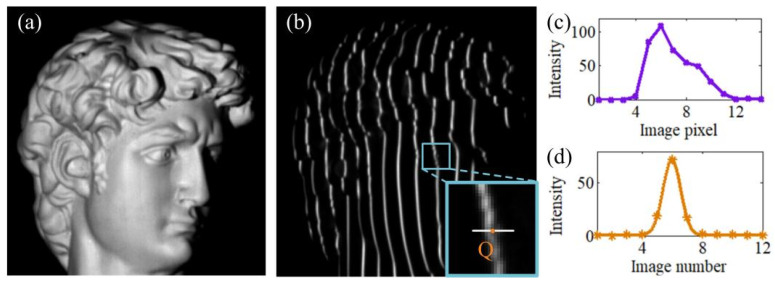
Time-domain Gaussian curve is unaffected by a complex object surface. (**a**) The plaster statue with a complex surface. (**b**) A complex surface is illuminated with Gaussian fringes (12 multi-line patterns are used to generate time-domain Gaussian curves). (**c**) Intensity profile along the white line in (**b**). (**d**) Time-domain Gaussian curves extracted from image pixel.

While the measuring object has a complex surface, the projected Gaussian fringes will be modulated by the surface and become severely deformed, as shown in Figure 6a,b. In line-structured light [15] or multi-line shift profilometry [16], the 3D point cloud is achieved by finding the peak positions of the spatial distribution of the Gaussian fringes. The severely deformed Gaussian fringes will make it extremely difficult to obtain accurate results of the complex surface.

**Figure 7:**
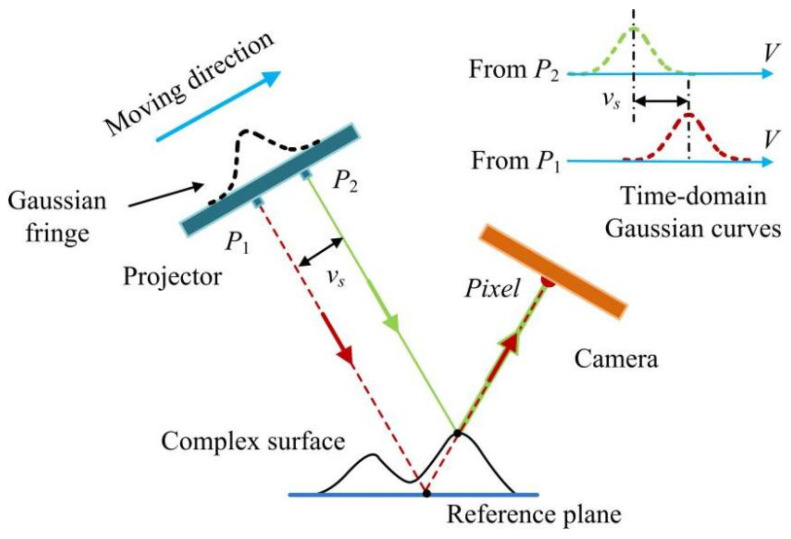
The influence of a complex surface on the time-domain Gaussian curves.

As illustrated in Figure 7, with respect to the complex surface, the camera pixels will receive the light emitted from the changed positions in the projector pattern. It will not deform the time–domain Gaussian curve, but will just cause extra shifting distance vs. (*x*, *y*) that seen in the time–domain Gaussian curve, *G_s_*, which can be expressed as the following:

(12) $$G_{s}\left(\lambda ,\eta ,\sigma ,v_{p}^{\prime \prime };v\right)=\lambda +\eta *\exp\left\{-\frac{{\left[v-\left(v_{p}+v_{s}\left(x,y\right)\right)\right]}^{2}}{2\sigma^{2}}\right\}$$

where $v_{p}^{\prime \prime }$ is the peak position of the time–domain Gaussian curve, *G_s_*.

It is shown in Figure 6c,d that, despite the projected Gaussian fringes being severely deformed on the complex surface, the extracted time–domain Gaussian curves still have an ideal shape. This characteristic of the time–domain Gaussian curves is helpful to compute accurate peak positions. Therefore, the proposed method is suitable to measuring a complex surface.

## 5. Experiments

Experiments have been carried out to verify the performance of our proposed method. A homemade FPP system, which consists of a DLP projector (LightCrafter 4500, Wintech, Beijing, China) and a CCD camera (MER-050-560U3M, Daheng, Beijng, China) with 8 mm lens (Computar, M0814-MP2, CBC Corporation, Tokyo, Japan), is applied to implement experiments. The captured images are processed using the MATLAB software (2012a). Two plaster statues (with the height of about 150 mm) and several planar targets are taken as the experimental subjects. The complementary gray-code unwrapping method [20] is applied in this paper to achieve the absolute projector column coordinates. And the calibrated third-order polynomial model [21] is used then to convert the absolute projector column coordinates into the height values.

**Figure 8:**
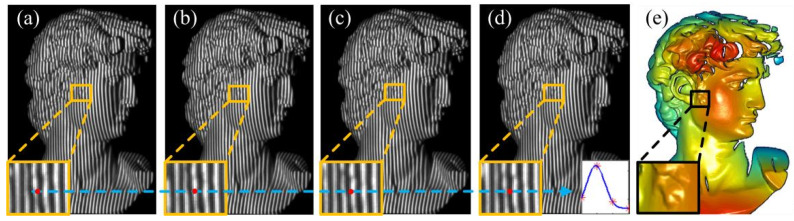
Projector coordinates are calculated with time-domain Gaussian curves (four shifting steps) and the Levenberg–Marquardt algorithm. (**a**–**d**) Gaussian fringes with different shifting distance (0, 1, 2, and 3 columns in projector plane) are projected onto the plaster statue, respectively. (**e**) The 3D reconstruction results.

In the first experiment, the performance of the proposed method is tested with the minimum shifting step (*n* = 4) and the minimum distance of shifting step (one column in projector plane, d*v* = 1). The distance between the two adjacent lines is four columns in the projector plane. The projector coordinates are computed with the Levenberg–Marquardt algorithm and Equation (1). During the experiment, four multi-line patterns are sequentially projected onto a plaster statue, and fringe images are captured simultaneously (see Figure 8a–d). It can be seen from the 3D reconstruction result (Figure 8e) that the proposed method can acquire a crowded and smooth point cloud of a complex surface, which proves that this method is suitable for measuring complex surfaces.

Although accurate projector coordinates can be achieved by using the Levenberg–Marquardt algorithm, it has a low calculation efficiency. In this experiment, 587 s are required to compute projector coordinates. The low calculation efficiency may result in the inability to make timely use of rapidly acquired 3D point cloud data.

**Figure 9:**
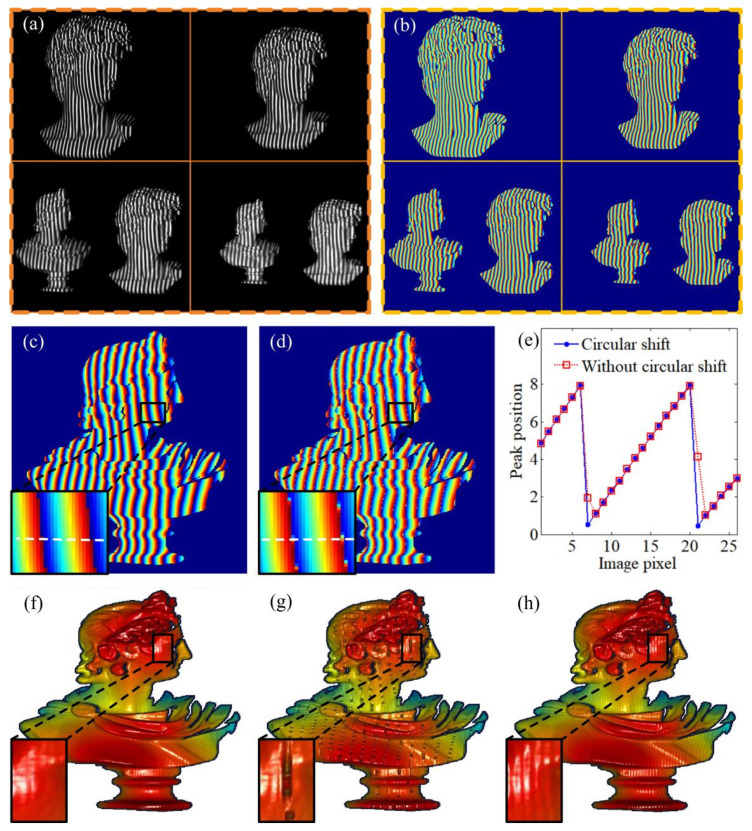
Computing projector coordinates with neural network. (**a**) The input part of training data. (**b**) The output part of training data. (**c**) Computing result is achieved using circular shift. (**d**) Computing result is achieved without using circular shift. (**e**) The fluctuation of peak positions along the white lines in (**c**,**d**). (**f**–**h**) The 3D reconstruction results of the neural network model using circular shift, the neural network model without using circular shift, and the Levenberg–Marquardt algorithm, respectively (step distance being 2 column in projector plane).

By contrast, a neural network can rapidly yield projector coordinates. In our work, the numbers of neurons in the input layer and hidden layer are four (*n* = 4) and six (*q* = 6), respectively. The activation function of *Tansig* is applied in the input layer, output layer, and hidden layer. The plaster statues are placed in different depths, and are sequentially illuminated by four multi-line patterns with a larger distance of shifting step (two columns in the projector plane, d*v* = 2). The distance between the two adjacent lines is eight columns in the projector plane. The input part (time–domain Gaussian curves) of training data can be extracted from the simultaneously captured fringe images (see Figure 9a). The results of the Levenberg–Marquardt algorithm are computed with eight multi-line patterns (the shifting steps are eight in number and the distance of a shifting step is one column in the projector) and set as the output part of the training data, as shown in Figure 9b.

It is demonstrated from Figure 9d,e,g that, while training data are preprocessed without circular shift, the trained neural network tends to smooth the mutation of peak positions in edge region and thus yields inaccurate results. Comparatively, these inaccurate peak positions in the edge region can be effectively avoided by adding circular shift in the preprocessing procedures (see Figure 9c).

As shown in Figure 9h, when the step distance becomes larger (two columns in the projector plane), the periodic error also can be found in the result of the Levenberg–Marquardt algorithm (with four shifting steps). In comparison, the periodic error can be greatly reduced in the result of the trained neural network (Figure 9f). Most importantly, by using the neural network technique, the computing time can be decreased significantly (from 587s to 11 ms), which may meet the requirements for real-time measurement or detection.

**Figure 10:**
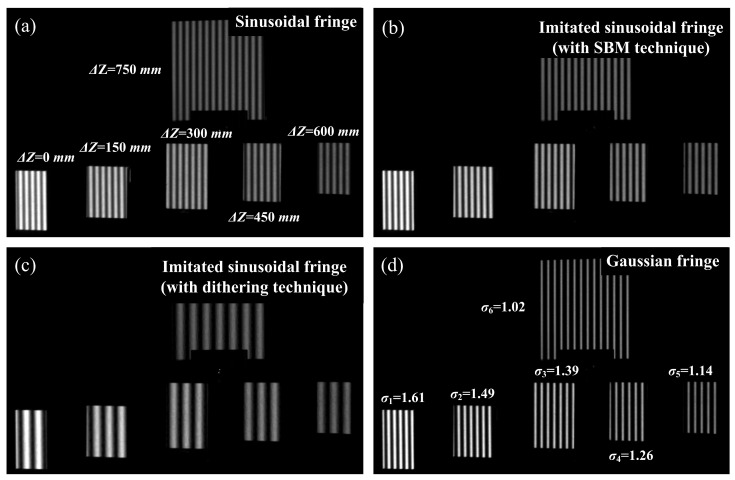
Testing the sensitivity of defocusing degree with multiple planar targets which are evenly placed from 0 mm to 750 mm. (**a**) Planar targets are illuminated with sinusoidal fringes. (**b**,**c**) Planar targets are illuminated with imitated sinusoidal fringes, which are generated using the SBM technique and dithering technique, respectively. (**d**) Planar targets are illuminated with Gaussian fringes.

Finally, the sensitivity to defocusing degree is tested, with several planar targets which are evenly placed from 0 mm to 750 mm (the interval is about 150 mm), as shown in Figure 10. For comparison, the sinusoidal patterns and the imitated sinusoidal patterns, which are generated using SBM technique and dithering technique, respectively, are applied in this experiment. The identical shifting step (four shifting steps) is applied, and the same fringe interval is used to generate a sinusoidal pattern, imitated sinusoidal pattern (SBM), and multi-line pattern in our proposed method (eight columns in the projector plane). A bigger fringe interval (16 columns in the projector plane) is used in the dithering technique. Due to the large depth between the planar targets (750 mm), the blur radius of the Gaussian fringes is also remarkably varied, from 1.61 (*σ*_1_ = 1.61) to 1.02 (*σ*_6_ = 1.02) (see Figure 10d).

**Figure 11:**
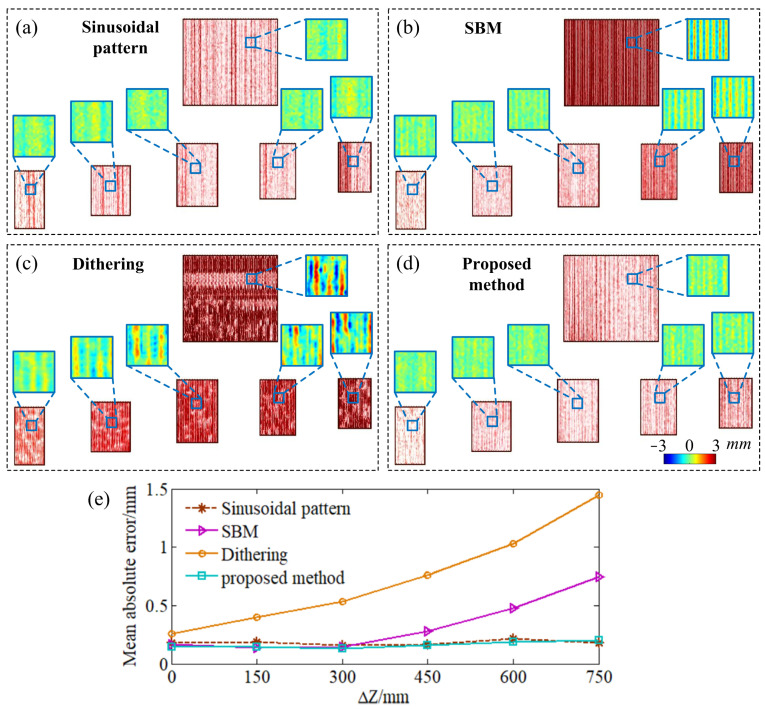
Comparison of the sensitivity to defocusing degree. (**a**–**d**) The 3D reconstruction results of sinusoidal pattern, SBM technique, dithering technique, and our proposed method. (**e**) The mean absolute errors in different depths.

The 3D reconstruction result of phase-shifting algorithm with 16 shifting steps is achieved and taken as the reference to calculate the 3D reconstruction errors of the different methods. The mean absolute errors (the average absolute value of the 3D reconstruction errors) are computed for comparison between the 3D reconstruction errors. With the varied defocusing degrees, the 3D reconstruction error of the phase-shifting algorithm with a sinusoidal pattern stays at a low level (Figure 11a,e). In comparison, the periodic errors in the reconstructed results of the SBM technique and the dithering technique increase rapidly (Figure 11b,c,e). It should be noted that the much larger error in the dithering technique just means that a greater defocusing degree is required by this technique. Comparatively, the proposed method shows much lower sensitivity to the varied defocusing degree (as shown in Figure 11d,e), and the periodic error can be suppressed without sacrificing acquisition speed. It is obvious that, with the same shifting steps, our proposed method has much greater DoF (about 750 mm) than that of the SBM technique (300 mm). The mean absolute errors are summarized in Table 1.

In this experiment, thirteen projector patterns (four multi-line patterns and nine gray-code patterns) are projected to achieve a 3D reconstruction result of the proposed method. Corresponding images are captured with a framerate of 400 Hz. The acquisition time of the 3D scan is 32.5 ms. By calculating the projector coordinates with the neural network model and by converting it into 3D coordinates with the polynomial reconstruction model, the computing time of the proposed method is squeezed into 35 ms (including 9 ms for computing projector coordinates, 11 ms for coordinate unwrapping, and 15 ms for calculating 3D coordinates). By contrast, it is shorter than that of the 3D scanning technique using the phase-shifting algorithm (358 ms). 

With respect to the measurement accuracy of the 3D scanning technique, the reflectivity of the object surface is also an important influencing factor. Actually, the non-uniform reflectivity will lead to obvious errors in the computed peak positions. Its generation mechanism and compensation method need to be further studied.

## 6. Conclusions

In this paper, a time-domain Gaussian fitting method is proposed to achieve a fast scanning speed and large DoF. The principle of determining projector coordinates with time-domain Gaussian curves is firstly put forward. By computing projector coordinates with a neural network, the proposed method has much lower sensitivity to the varied defocusing degree. The DoF of 3D scanning can be extended from 150 mm to 750 mm. Moreover, our proposed method not only can achieve a high speed projection of Gaussian fringes, but the computing time also can be reduced dramatically from 587 s to 11 ms. With these advantages, our proposed method can be used for measuring large-scale parts in real-time.

## Figures and Tables

**Table 1:** Comparison of mean absolute errors in the 3D reconstructed results (unit: mm).

	Relative Depth (ΔZ)					
	0 mm	150 mm	300 mm	450 mm	600 mm	750 mm
Sinusoidal pattern	0.177	0.187	0.158	0.165	0.213	0.177
SBM	0.167	0.135	0.147	0.276	0.480	0.746
Dithering	0.256	0.397	0.534	0.764	1.026	1.450
Our method	0.145	0.142	0.130	0.161	0.190	0.201

## Data Availability

Data will be made available on request.
